# Deproteinization of four macroporous resins for rapeseed meal polysaccharides

**DOI:** 10.1002/fsn3.1309

**Published:** 2019-12-05

**Authors:** Shengzhou Duan, Qian Huang, Xiaoqian Shen, Jie Hu, Xiangzhou Yi, Zhenshun Li, Baomiao Ding

**Affiliations:** ^1^ College of Life Science Yangtze University Jingzhou China; ^2^ Jingchu Food Research & Development Centre Yangtze University Jingzhou China

**Keywords:** deproteinization, macroporous resin, polysaccharide, purification, rapeseed meal

## Abstract

In this study, the adsorption/desorption characteristics of rapeseed meal polysaccharides extract on four resins (HP‐20, D3520, XAD‐16, and AB‐8) were evaluated. The results indicated that HP‐20 resin had the best purification effect. Based on static adsorption test, the kinetics and isotherms of the four resins for protein and polysaccharide were investigated. The adsorption test showed that the pseudo‐second‐order kinetics model and the Freundlich isotherm model were more suitable for explanation of the adsorption process for protein and polysaccharide. Static desorption test showed that the highest protein desorption ratios of HP‐20, D3520, and AB‐8 resins could be obtained with 60% ethanol solution as eluate, and the highest protein desorption ratios of XAD‐16 resin could be obtained with 40% ethanol solution as eluate. Dynamic adsorption/desorption tests of HP‐20 resin showed that the deproteinization ratio was 91% and the polysaccharide recovery ratio was 62% when the treatment amount was 1.5 BV. Compared with three traditional methods, HP‐20 resin adsorption method that the deproteinization ratio was 82% was more potent than the three traditional methods for purifying polysaccharides from rapeseed meal. In addition, UV/vis spectroscopy showed that most of the protein was absorbed by resins, and FT‐IR spectroscopy indicated that the purity of the polysaccharide after purification was improved. Rapeseed meal polysaccharides could be effectively deproteinized using HP‐20 resin, and it was suitable for purifying polysaccharides from rapeseed meal.

## Graphical abstract

In this study, the purification of rapeseed meal polysaccharides by macroporous resin was studied. HP‐20 resin had the better purification effect.

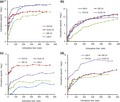

## 1 INTRODUCTION

At present, 3.5 million tons of rapeseed oil is produced each year in China; meanwhile, 5.3 million tons of rapeseed meal is abandoned. The rapeseed meal left after extracting rapeseed oil was rich in polysaccharide, proteins, carbohydrates, polyphenols, and minerals (Dossou et al., 2018; Sun, Qi, Qi, Xu, Juan, & Zhe, 2011; Tan, Mailer, Mailer, Blanchard, & Agboola, 2011). The polysaccharides isolated from natural sources usually show various significant bioactivities, such as anti‐inflammatory, antitumor, and immunomodulatory effects, which are strongly affected by their chemical structures and chain conformations. The polysaccharides from Ixeris polycephala could enhance phagocytosis of macrophages (Luo et al., 2018). The brown seaweed Sargassum wightii polysaccharides significantly reduced the proliferation of breast cancer cells (Vaikundamoorthy, Krishnamoorthy, Krishnamoorthy, Vilwanathan, & Rajendran, 2018). The polysaccharides from mulberry could decrease the blood glucose levels of hyperglycemia mice (Liao et al., 2018). The P. haitanensis polysaccharides showed good antioxidant capacity (Cai et al., 2017). Rapeseed polysaccharides have been extracted using water or 5% NaOH aqueous solution (Zhu & Wu, 2009), but the polysaccharides extracted by these methods often contain some proteins. Traditionally, the Sevage method, the trichloroacetic acid method, and active carbon adsorption were used to remove proteins in the solution. And these methods often consume a lot of manpower and material resources, resulting in large loss of polysaccharides and low deproteinization ratio (Yang et al., 2012; Zhu & Wu, 2009).

**Figure 1:**
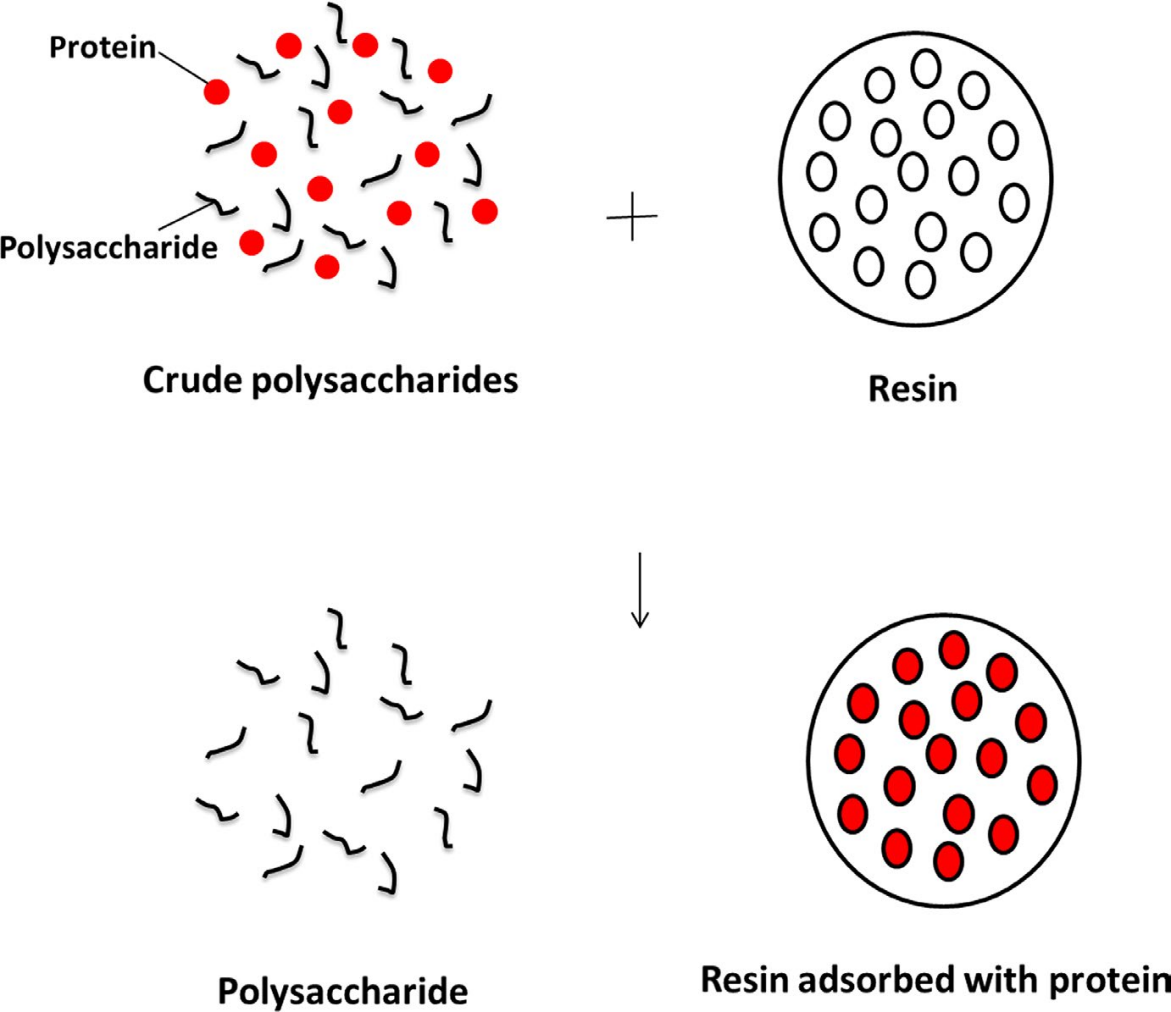
The schema of adsorption mechanism

Macroporous resin has advantages of high recovery ratio, good selectivity, mild adsorption conditions, low operating cost, and easy regeneration (Charpe & Rathod, 2015; Yang, Zhao, Zhao, & Lin, 2016; Zou et al., 2015). The major advantages of macroporous resins can obtain higher purity natural products, providing great convenience for the study of natural products (Chen et al., 2016; Liu et al., 2010; Sheng, Tingting, Tingting, Xuanying, Xiangxiang, & Mengdi, 2016; Xue, Xu, Xu, Lu, Ju, & Xing, 2016; Yang et al., 2012; Zhuang et al., 2016). In recent years, macroporous resins have been used to purify bioactive components in natural products. To our knowledge, little research was carried out the performance and separation characteristics of macroporous resins for purifying polysaccharides from rapeseed meal. Figure 1 shows the schema of adsorption mechanism (Liu, Hua, Hua, Wang, & Yang, 2019; Sun et al., 2017). The purpose of this work was to research the adsorption and desorption behaviors, kinetics and isotherms of HP‐20, D3520, XAD‐16, and AB‐8 resins on the polysaccharides from rapeseed meal. Then, the dynamic adsorption and desorption tests of the selected resin were carried out. The purification effect between the selected resin and three other traditional purification techniques was compared, including the use of trichloroacetic acid, Sevage reagent, and activated carbon. In addition, the spectral properties of the polysaccharides before and after adsorption were determined to test and verify the adsorption effect.

## 2 MATERIALS AND METHODS

### 2.1 Adsorbents and chemicals

HP‐20, D3520, XAD‐16, and AB‐8 resins were purchased from Yuanye Biotechnology Co., Ltd. The structural parameters of these resins are shown in Table 1. Rapeseed meal (Zhongyouza 12) was purchased from Tianjiao Biological Engineering Development Co., Ltd.. All other chemicals were of analytical grade and were purchased from Sinopharm Chemical Reagent Co., Ltd.

**Table 1:** Physical and chemical properties of the macroporous resins

Type	Polarity	Particle diameter (mm)	Polymeric matrix	Surface area (m^2^/g^1^)	Average pore diameter (nm)
D3520	Nonpolar	0.30 − 1.25	Styrene	480 − 520	8.5 − 9.0
AB−8	Weak polar	0.30 − 1.25	Styrene	480 − 520	13.0 − 14.0
HP−20	Nonpolar	0.30 − 1.25	Styrene	600 − 650	24.0 − 26.0
XAD−16	Nonpolar	0.70	Divinylbenzene	800	15.0

### 2.2 Pretreatment of resins and samples

According to the method of Zhuang, et al with some modifications (Zhuang et al., 2016), the resins were pretreated to remove the impurity during resin producing. First, the resins were soaked in 95% ethanol for 24 hr and then washed with deionized water to remove ethanol. Second, the resins were immersed in 1 M NaOH and 1 M HCl for 5 hr, respectively. Finally, they were washed with deionized water until the pH of the resins was 7, and the resins were dried at 60°C under reduced pressure. The resins were soaked in 95% ethanol overnight before use and then washed with deionized water to remove the ethanol.

Rapeseed meal was smashed into powder using high‐speed smashing machines (Tianjin Hengrui Science Instruments Co., Ltd). The fat in rapeseed meal was removed using the method described by Zhu & Wu with some modifications (Zhu & Wu, 2009). Defatted rapeseed meal was twice extracted with 80% (v/v) ethanol (1 g/20 ml) at 80°C for 2 hr to remove the phenolic compounds and oligosaccharides (decoloration). The high concentration crude polysaccharides (HC‐CPS) were produced by the defatted and decolored rapeseed meal, and extracted with water (1 g/28 ml) at 94°C for 3 hr. The low concentration crude polysaccharides (LC‐CPS) were obtained by the defatted and decolored rapeseed meal, and thrice extracted with water (1 g/28 ml) at 94°C for 3 hr and mixed.

### 2.3 Adsorption kinetics of four resins

According to the method of Chen, et al with some modifications (Sheng et al., 2016), pretreated resin (3.0 g) was mixed with 100 ml HC‐CPS and LC‐CPS in flasks, respectively. And each flask was placed in a water‐bath shaker (170 rpm) at 25°C. Five hundred microliters of solution in each flask was withdrawn at 4, 8, 12, 16, 20, 25, 30, 35, 40, 50, 60, 70, 80, 100, 120, 140,160, 190, 220, 250, 280, 320, 360, 400, 440, 490, 540, and 590 min, respectively. The concentration of proteins was determined using bovine serum albumin as standard, and the concentration of polysaccharides was measured by the phenol − sulfuric acid method using glucose as standard. The adsorption kinetics was evaluated by pseudo‐first‐order model and pseudo‐second‐order model, and the mechanism involved in predicting the adsorption process was tested.

### 2.4 Desorption tests of four resins

According to the method of Zhuang et al. (2016) with some modifications, each pretreated resin (1 g) mixed with 50 ml HC‐CPS in 100 ml flasks. And each flask was placed in a water‐bath shaker (170 rpm) at 25°C. After adsorption equilibrium, the resins were washed with deionized water for three times and then desorbed with 50 ml different concentrations of ethanol (0%, 20%, 40%, 60%, 80%, and 100% ethanol content) in the 100 ml flasks, respectively. All these flasks were sealed and placed in a water‐bath shaker (170 rpm) at 25°C for 12 hr.

### 2.5 Adsorption isotherms of four resins

Adsorption isotherms were examined according to the method of Yang et al with some modifications (Yang et al., 2015). The HC‐CPS was diluted with zero fold, one fold, two fold, three fold, and four fold of deionized water, respectively. Then, pretreated resin (1 g) was poured into 100 ml flask, and 50 ml rapeseed meal polysaccharide was added, respectively. All the flasks were sealed and shaken using a water‐bath shaker (170 rpm) at 25°C for 10 hr. Two standard theoretical models, Langmuir and Freundlich models were used to describe the adsorption behavior.

### 2.6 Dynamic adsorption and desorption tests on HP‐20 resin

A glass column (16mm × 400 mm) was used in the dynamic adsorption and desorption experiments, which was wet‐packed with HP‐20 resin (bed volume, BV = 20 ml) (Zou et al., 2015).The dynamic breakthrough experiments were carried out at 25°C. In dynamic adsorption test, the rapeseed meal polysaccharides solution was loaded continuously on the glass column at a flow rate of 1 ml/min. In dynamic desorption test, the column was first washed with deionized water and then eluted with ethanol–water solutions at volumetric ratios of 60/40, at a flow rate of 1ml/min. The concentration of protein and polysaccharide was determined during adsorption and desorption tests.

### 2.7 Purification of polysaccharide for three traditional methods

The polysaccharide was purified by trichloroacetic acid, Sevage and activated carbon. 30 ml LC‐CPS and 30 ml of 20% trichloroacetic acid were mixed and stirred for 30 min, and the fluid was centrifuged at 4,000 *g* for 20 min to remove precipitate. 10 ml LC‐CPS and 40 ml Sevage reagent (n‐BuOH/CHCl_3_, v/v = 1:4) were mixed and stirred for 30 min, and the fluid was centrifuged at 4000g for 20 min. The supernatant was taken and the operation was repeated three times. Active carbon (1 g) mixed with 50 ml LC‐CPS in 100 ml flasks. And the flask was placed in a water‐bath shaker (170 rpm) at 25°C for 10 hr, then filtering to remove active carbon. For all these three traditional purification methods, the concentration of protein and polysaccharide was determined.

### 2.8 Characterization of polysaccharide before and after adsorption

The polysaccharides solution before and after treatment was characterized by UV/vis spectroscopy and Fourier transform infrared (FT‐IR) spectroscopy. The UV/vis wave spectra (200 − 600 nm) of the polysaccharides solution before and after adsorption were determined by an UV/vis 2600 spectrophotometer (SHIMADZU, Kyoto, Japan) (Yang et al., 2012). The polysaccharide samples before and after adsorption were freeze‐dried and mixed with KBr at the ratio of 1:100 (w/w). The FT‐IR spectra were measured using a Nicolet Nexus 470 Fourier transform spectrometer (Madison, USA) in a range of 4000–400 cm^−1^.

### 2.9 The equations used in this study

According to previous reports (Das, Goud, & Das, 2018; Ho & Ofomaja, 2005; Li et al., 2018; Sheng et al., 2016) with some modifications, the equations were expressed as follows:

Adsorption ratio:

(1) $$A\left(\%\right)=\frac{\left(C_{0}-C_{e}\right)}{C_{0}}\times 100\%$$

Desorption ratio:

(2) $$D\left(\%\right)=\frac{C_{d}\times V_{d}}{\left(C_{0}-C_{e}\right)V_{i}}\times 100\%$$

Adsorption capacity:

(3) $$q_{t}=\frac{\left(C_{0}-C_{t}\right)V_{i}}{W}\times 100\%$$

The pseudo‐first‐order kinetics model:

(4) $$\ln\left(q_{e}-q_{t}\right)=-k_{1}t+\ln q_{e}$$

The pseudo‐second‐order kinetics model:

(5) $$\frac{t}{q_{t}}=\frac{1}{q_{e}}t+\frac{1}{k_{2}q_{e}^{2}}$$

The particle diffusion kinetics model:

(6) $$q_{t}=k_{d}\cdot t^{1/2}+C$$

The Langmuir equation and its variable form:

(7) $$q_{e}=\frac{q_{m}K_{L}C_{e}}{1+K_{L}C_{e}}\frac{C_{e}}{q_{e}}=\frac{1}{q_{m}}\cdot C_{e}+\frac{1}{q_{m}K_{L}}$$

The Freundlich equation and its variable form:

(8) $$q_{e}=K_{F}C_{e}^{1/n}\;\ln q_{e}=\frac{1}{n}\cdot \ln C_{e}+\ln K_{F}$$

Deproteinization ratio:

(9) $$\text{DR}\left(\%\right)=\frac{P_{b}-P_{a}}{P_{b}}\times 100\%$$

Polysaccharide recovery ratio:

(10) $$\text{PR}\left(\%\right)=\frac{H_{a}}{H_{b}}\times 100\%$$

where *A* and *D* are the adsorption ratio (%) and desorption ratio (%), respectively. *C_0_* and *C_t_* denote the concentration (mg/ml) of protein and polysaccharide at initial absorbent solution and at time *t*, respectively. *C_e_* and *C_d_* denote the concentration (mg/ml) of protein and polysaccharide at equilibrium stage and at desorption solution. *V_i_* and *V_d_* are the volume (ml) of adsorbent solution and the desorption solution. *W* stand for the macroporous resin weight (g). *q_e_* and *q_t_* are the adsorption capacities protein and polysaccharide at equilibrium stage and time *t*, respectively. *q_m_* stand for the maximum adsorption capacity. *k_1_*, *k_2,_* and *k_d_* denote the pseudo‐first‐order rate constant, the pseudo‐second‐order rate constant, and particle diffusion kinetics rate constant. *C* is the constant in particle diffusion kinetics model. *K_L_* is the Langmuir constant. *K_F_* is the Freundlich constant, and *1/n* is an empirical constant related to the magnitude of the adsorption driving force. *DR* is deproteinization ratio. *P_b_* and *P_a_* stand for the concentrations of protein (mg/ml) before and after treatment, respectively. *PR* is polysaccharide recovery ratio. *H_b_* and *H_a_* stand for the concentrations of polysaccharide (mg/ml) before and after treatment, respectively.

## 3 RESULTS AND DISCUSSION

### 3.1 Adsorption kinetics on four resins

**Figure 2:**
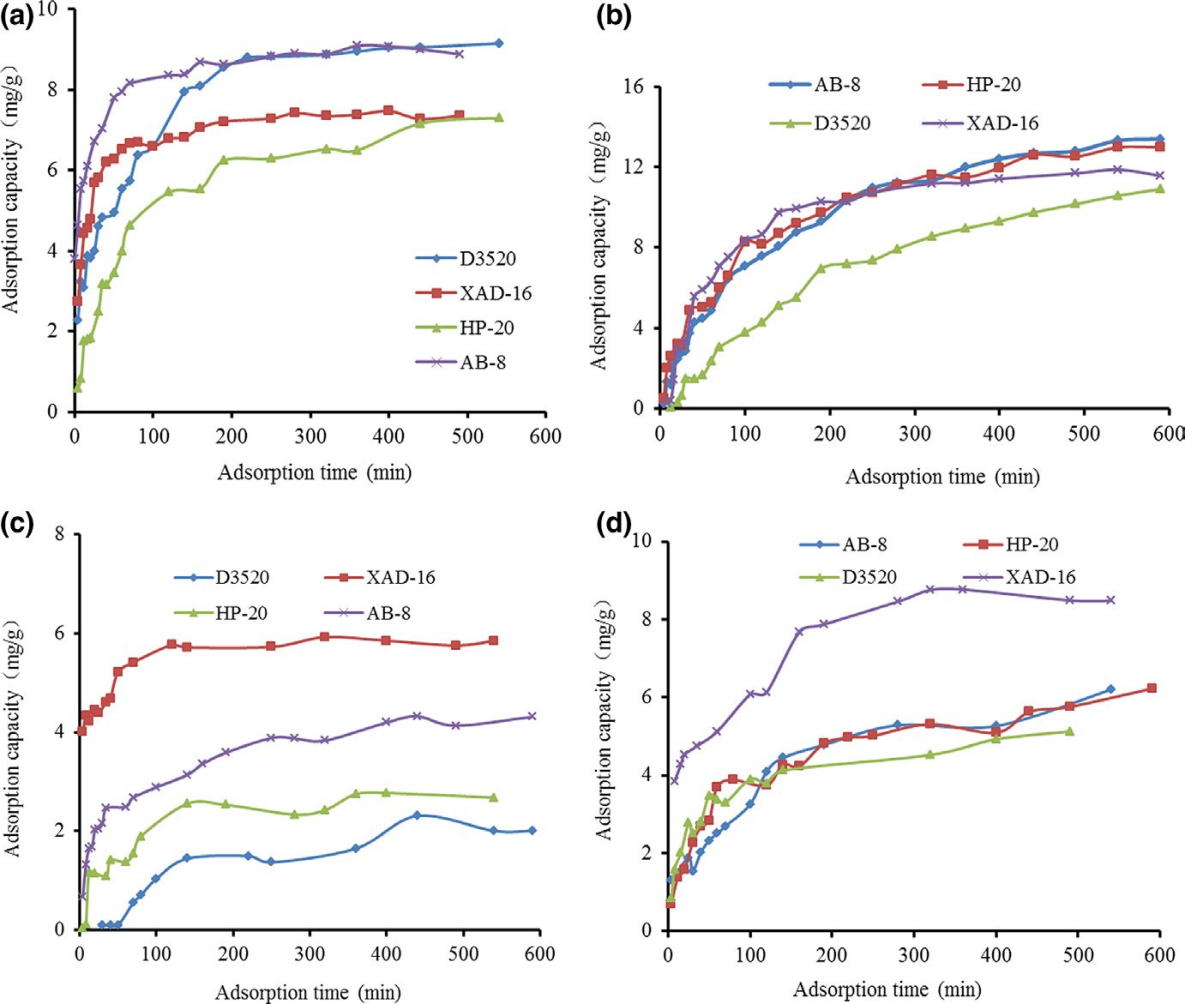
Adsorption kinetics curves

Figure 2 shows the adsorption kinetics curves of four resins. The concentration of protein was 0.195 mg/ml, and the concentration of polysaccharide was 0.416 mg/ml in LC‐CPS. The concentration of protein was 0.320 mg/ml, and the concentration of polysaccharide was 0.845 mg/ml in HC‐CPS. The protein adsorption capacities of the four resins increased rapidly at the beginning and then slightly increased until adsorption equilibrium, and the adsorption capacities of polysaccharide had similar features. This phenomenon was similar to using macroporous resin to enrich antioxidant components in garlic (Zou, Zhao, Zhao, Yang, Lin, & Wang, 2017). The protein adsorption ratios of HP‐20, D‐3520, XAD‐16, and AB‐8 resins were about 82%, 76%, 83%, and 91% in LC‐CPS at the adsorption equilibrium, respectively. The polysaccharide adsorption ratios of HP‐20, D‐3520, XAD‐16, and AB‐8 resins were about 13%, 12%, 28%, and 20% in LC‐CPS at the adsorption equilibrium, respectively. The protein adsorption ratios of HP‐20, D‐3520, XAD‐16, and AB‐8 resins were about 79%, 62%, 76%, and 75% in HC‐CPS at the adsorption equilibrium, respectively. The polysaccharide adsorption ratios of HP‐20, D‐3520, XAD‐16, and AB‐8 resins were about 15%, 10%, 18%, and 15% in HC‐CPS at the adsorption equilibrium, respectively. The results indicated that the adsorption ratios of the four resins on protein and polysaccharide decreased when the concentration of polysaccharides increased, but the adsorption ratios of HP‐20 on the protein were higher than other three resins. Therefore, HP‐20 resin was more suitable for deproteinization of rapeseed meal polysaccharides.

Pseudo‐first‐order model, pseudo‐second‐order model, and particle diffusion kinetics model were used to evaluate the adsorption processes in order to research the adsorption mechanisms of the four resins (Guo et al., 2017). As shown in Tables 2, 3, 4, the correlation coefficient of the pseudo‐second‐order model was higher than the pseudo‐first‐order model. And the calculated *q*_te_ (theoretical equilibrium adsorption capacity calculated by kinetic model) was closer to the experimental *q*_e_ for protein and polysaccharide in LC‐CPS. Therefore, the pseudo‐second‐order model could better explain the adsorption process for the four resins in LC‐CPS. The pseudo‐second‐order model had a higher correlation coefficient than the pseudo‐first‐order model of the protein, but the q_te_ calculated by the pseudo‐first‐order model was more consistent with the experimental q_e_ in HC‐CPS. This might be due to the polysaccharide molecules occupied the adsorption site on the resin, resulting in a lower adsorption capacity than the theoretical value. This phenomenon was more obvious when the concentration of polysaccharide increased. As shown in Table 4a,b, the plots of *q*_t_ versus *t* ^1/2^ were not linear, indicating that the adsorption process was controlled by the internal diffusion of particles and the external diffusion of particles (Zhuang et al., 2016). Therefore, the pseudo‐second‐order kinetics model was more suitable for describing the adsorption process of four resins on rapeseed meal polysaccharides. This result was similar to the separation of chlorogenic acid from Eupatorium adenophorum extract by macroporous resin (Liu et al., 2016).

**Table 2:** Values of parameters pseudo‐first‐order kinetic model of (a) protein (b) polysaccharide

(a) Resin		Protein of LC‐CPS				Protein of HC‐CPS		
	*q*_e_	*R* ^2^	*q*_te_	*k*_1_	*q*_e_	*R* ^2^	*q*_te_	*k*_1_
AB−8	8.966	0.930	3.2785	0.0116	12.764	0.939	13.4961	0.0088
D3520	9.143	0.967	6.0982	0.0101	9.942	0.956	12.1472	0.0074
HP−20	7.086	0.931	5.469	0.0077	12.305	0.987	11.1295	0.0081
XAD−16	7.407	0.851	2.2052	0.0094	11.413	0.815	11.0928	0.0128

(b) Resin		Polysaccharide of LC‐CPS				Polysaccharide of HC‐CPS		
	*q*_e_	*R* ^2^	*q*_te_	*k*_1_	*q*_e_	*R* ^2^	*q*_te_	*k*_1_
AB−8	4.363	0.913	2.9612	0.0068	5.347	0.634	3.4542	0.0062
D3520	2.226	0.660	2.4481	0.0043	4.755	0.524	2.4298	0.0038
HP−20	2.710	0.647	1.7766	0.0054	5.870	0.933	4.4256	0.0065
XAD−16	5.749	0.647	1.2371	0.0094	8.181	0.807	4.3004	0.0075

**Table 3:** Values of parameters pseudo‐second‐order kinetic model of (a) protein (b) polysaccharide

(a) Resin		Protein of LC‐CPS				Protein of HC‐CPS		
	*q*_e_	*R* ^2^	*q*_te_	*k*_2_	*q*_e_	*R* ^2^	*q*_te_	*k*_2_
AB−8	8.966	0.999	9.3197	0.0086	12.764	0.980	16.4474	0.0005
D3520	9.143	0.997	9.7943	0.003	9.942	0.965	18.1818	0.0001
HP−20	7.086	0.991	8.3195	0.0017	12.305	0.991	14.9925	0.0007
XAD−16	7.407	0.999	7.6278	0.0116	11.413	0.997	12.9702	0.0013

(b) Resin		Polysaccharide of LC‐CPS				Polysaccharide of HC‐CPS		
	*q*_e_	*R* ^2^	*q*_te_	*k*_2_	*q*_e_	*R* ^2^	*q*_te_	*k*_2_
AB−8	4.363	0.980	4.6773	0.0045	5.347	0.924	6.1463	0.0023
D3520	2.226	0.731	2.2727	0.0083	4.755	0.913	4.8123	0.0052
HP−20	2.710	0.800	3.4188	0.0023	5.870	0.966	6.8027	0.0020
XAD−16	5.749	0.995	5.8962	0.0356	8.181	0.985	8.5911	0.0042

**Table 4:** Values of parameters particle diffusion kinetics model of (a) protein (b) polysaccharide

(a) Resin	Protein of LC‐CPS			Protein of HC‐CPS		
	*R* ^2^	*C*	*k*_d_	*R* ^2^	*C*	*k*_d_
AB−8	0.758	5.179	0.204	0.957	0.816	0.545
D3520	0.884	2.524	0.335	0.987	1.990	0.568
HP−20	0.907	0.612	0.315	0.947	1.003	0.560
XAD−16	0.697	4.494	0.164	0.845	2.316	0.470

(b) Resin	Polysaccharide of LC‐CPS			Polysaccharide of HC‐CPS		
	*R* ^2^	*C*	*k*_d_	*R* ^2^	*C*	*k*_d_
AB−8	0.919	1.208	0.146	0.843	1.273	0.196
D3520	0.882	0.970	0.151	0.696	1.425	0.159
HP−20	0.735	0.484	0.100	0.913	0.937	0.242
XAD−16	0.687	4.219	0.088	0.813	3.812	0.217

### 3.2 Adsorption and desorption capacity of four resins in HC‐CPS

The protein adsorption ratios of HP‐20, D‐3520, XAD‐16, and AB‐8 resins were about 79%, 62%, 76%, and 75% in HC‐CPS at the adsorption equilibrium, respectively. The polysaccharide adsorption ratios of HP‐20, D‐3520, XAD‐16, and AB‐8 resins were about 15%, 10%, 18%, and 15% in HC‐CPS at the adsorption equilibrium, respectively. The results showed that the protein and polysaccharide adsorption ratios of D3520 resin was lower than that of the other resins, and the adsorption ability of D3520 resin was weaker than that of the other three resins. This result might be due to the small average pore size of D3520 resin, which made it difficult for protein molecules to enter the resin. In addition, XAD‐16 resin had a larger surface area, which made it had stronger adsorption ability to polysaccharide than other resins (Yuanfeng et al., 2016).

**Figure 3:**
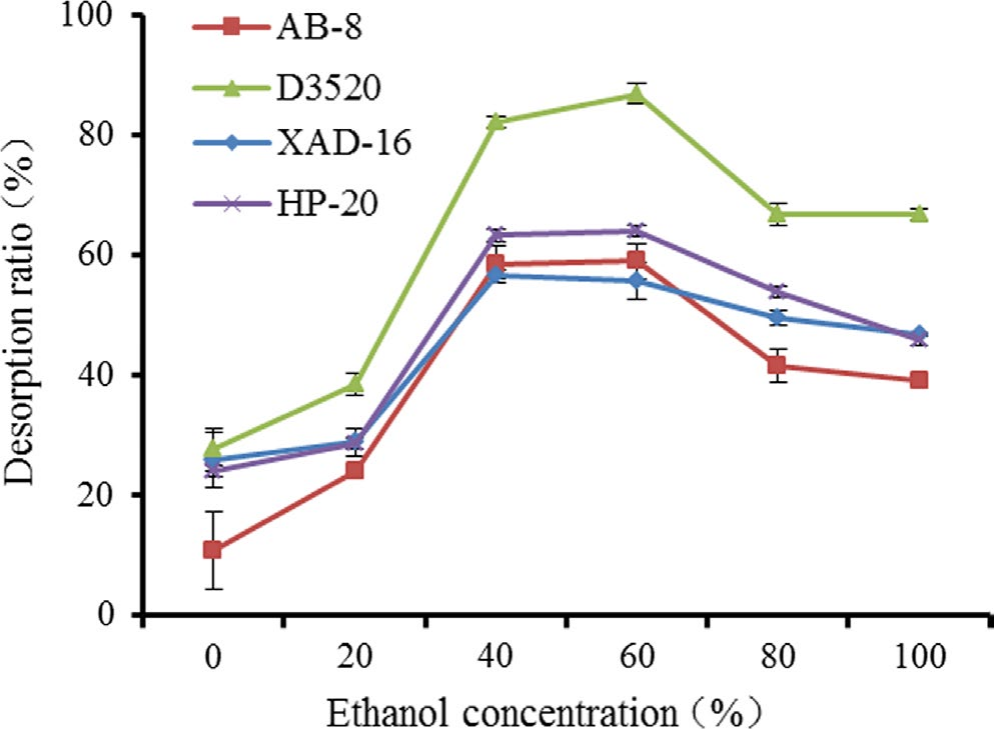
Desorption ratio of the protein on the different resins by ethanol of various concentrations

Figure 3 shows the protein desorption ratios with four resins in different ethanol concentrations. Based on the polarity matching principle, a solute is easy to dissolve in solvent with the similar polarity, namely, a nonpolar solute dissolves in a nonpolar solvent easily, while a polar solute can easily dissolve in a polar solvent (Yang et al., 2015). The polarity of protein is similar to that of 40%–60% ethanol. With the increase of ethanol concentration, protein desorption ratios increased at first and then decreased. This was similar to the results of Zhuang, et al using macroporous resin to purify the umami peptides from soy sauce (Chen et al., 2016; Zhuang et al., 2016). The highest protein desorption ratios of HP‐20, D3520, XAD‐16, and AB‐8 resins were 64%, 85%, 56%, and 59%, respectively. HP‐20, D3520, and AB‐8 resins had the highest protein desorption ratio at 60% ethanol concentration, but XAD‐16 resin had the highest protein desorption ratio at 40% ethanol concentration. The desorption ratio of D3520 resin was the highest, but it had lowest adsorption capacities of protein. The results showed that D3520 resin had a weaker proteins binding ability than the other three resins, which resulted in a low protein adsorption capacity and high desorption ratio.

### 3.3 Adsorption isotherms of the polysaccharide on four resins

Adsorption isotherms described the relationship between the equilibrium adsorption capacity and the equilibrium concentration of adsorbate at constant temperature (Chen et al., 2016). To establish an optimum model, the experimental equilibrium data were fitted to two isotherm models: Langmuir and Freundlich models. The adsorption isotherms of four resins are shown in Table 5. The Langmuir isotherm model described the monolayer adsorption onto a homogeneous surface with no interaction between adjacent adsorbed molecules. K_L_ was the Langmuir constant, the values of K_L_ indicated whether the isotherm was favorable (0 < K_L_ < 1), linear (K_L_ = 1), or unfavorable (K_L_ > 1). The Freundlich isotherm model reflected the multilayer adsorption process on a heterogeneous surface. K_F_ was a Freundlich affinity parameter for a hetero‐disperse system. The n was related to the magnitude, the sorption driving force, and the energy distribution of the sorption sites. Adsorption was easily to take place when the value of 1/*n* was between 0.1 and 0.5, but it was difficult to adsorption when the value of 1/*n* was between 0.5 and 1 (Chen et al., 2016).

**Table 5:** Parameters of Langmuir and Freundlich Models of (a) protein (b) polysaccharide

(a) Protein	*R* ^2^	*K*_L_	*q*_m_	*R* ^2^	*K*_F_	1/*n*
HP−20	0.922	43	‐	0.858	6.175	0.217
D3520	0.998	5,694	3.512	0.923	3.911	0.027
AB−8	0.943	492	4.065	0.840	6.193	0.143
XAD−16	0.898	886	5.643	0.793	9.416	0.145

(b) Polysaccharide	*R* ^2^	*K*_L_	*q*_m_	*R* ^2^	*K*_F_	1/*n*
HP−20	0.935	0.684	25.641	0.978	12.839	0.900
D3520	0.940	1.584	20.790	0.958	7.629	0.612
AB−8	0.880	0.570	20.325	0.957	9.276	0.946
XAD−16	0.973	1.151	21.551	0.921	11.172	0.620

The K_L_ values were all >1 when Langmuir model was used to fit the process of adsorbing protein by four resins, so the Langmuir model was not suitable for explaining the adsorption of protein by four resins. The correlation coefficients of the Freundlich model of HP‐20, D3520, and AB‐8 resins were higher than those of the Langmuir model, when Langmuir and Freundlich models were used to fit the process of explaining polysaccharide adsorption process on four resins. Meanwhile, the K_L_ values of D3520 and XAD‐16 resin was >1. Therefore, Freundlich model was more suitable for explaining the adsorption process of the four resins on the protein and polysaccharide. This result was consistent with that of Bao, Yuan et al. using macroporous resin to remove bitterness from citrus juice (Bao, Yuan, Yuan, Zhao, Liu, & Gao, 2015). The research showed that 0 < 1/*n* < 0.5 for four resins adsorbing protein and 0.5 < 1/*n* < 1 for four resins adsorbing polysaccharide. It indicated that the four resins had strong adsorption ability for protein and weak adsorption ability for polysaccharide. The four resins could be used for deproteinization of rapeseed meal polysaccharides.

### 3.4 Dynamic adsorption and desorption tests on HP‐20 resin in HC‐CPS

**Figure 4:**
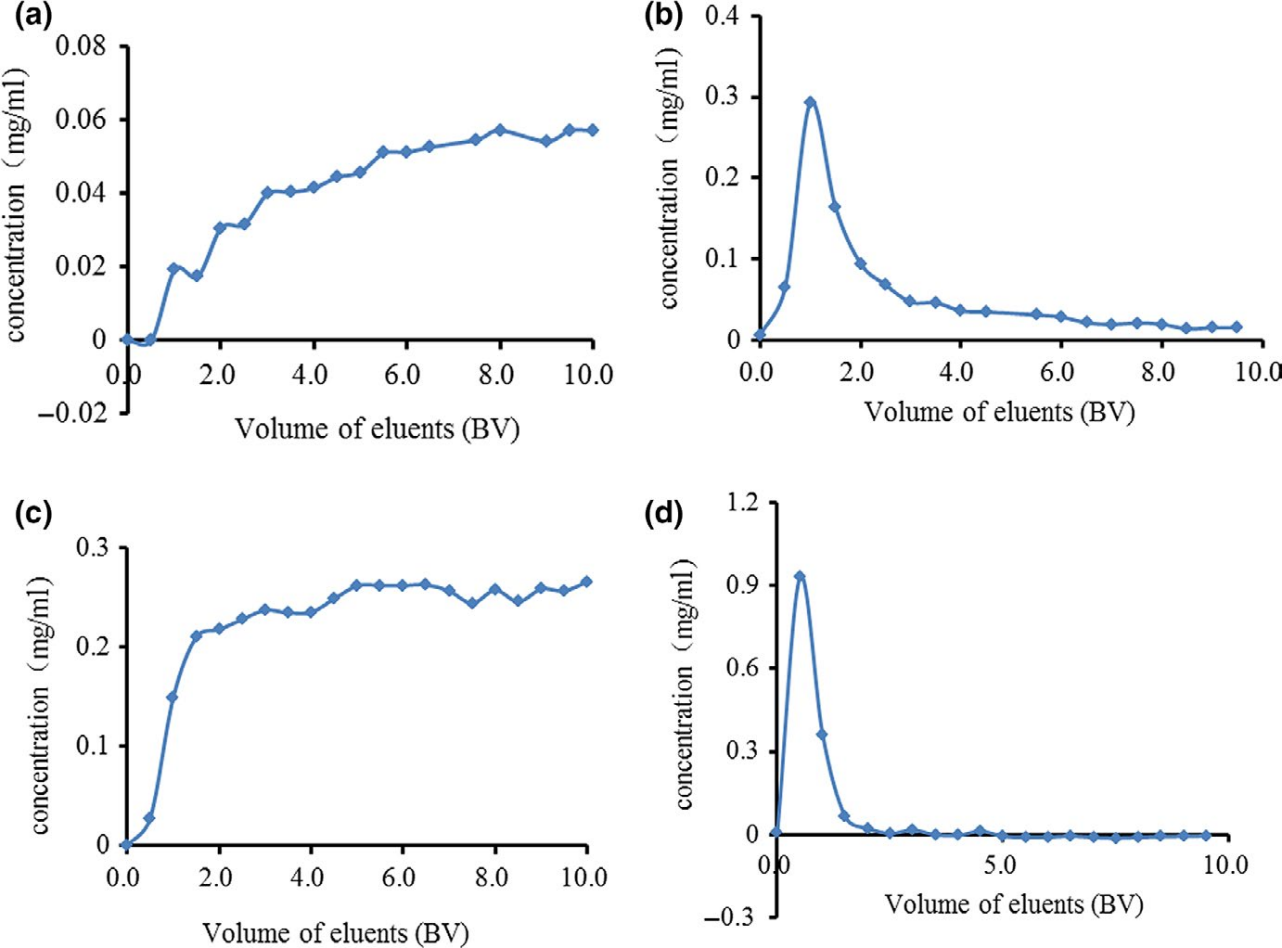
Dynamic adsorption curves and dynamic desorption curves of protein and polysaccharide on a column packed with HP‐20 resin

Breakthrough curve was an important feature in the adsorption process. It reflected the adsorption equilibrium relationship between the solute and resins. Considering that HP‐20 resin had the best deproteinization effect, HP‐20 resin was used to further research the dynamic adsorption and desorption of rapeseed meal polysaccharides. Figure 4 shows the dynamic adsorption and desorption characteristics of HP‐20 resin in HC‐CPS. The results showed that the protein was completely adsorbed by HP‐20 resin before 0.5 BV, and the deproteinization ratio was 91% at 1.5 BV. Then, the protein adsorption ratio gradually decreased and reached a steady plateau after 5.5 BV, and the deproteinization ratio was 73% at 5.5 BV. Meanwhile, the polysaccharide was almost completely adsorbed by HP‐20 resin before 0.5 BV, and polysaccharide recovery ratio was 62% at 1.5 BV. Then, the polysaccharide adsorption ratio gradually decreased and reached a stable steady plateau after 5.0 BV, and polysaccharide recovery ratio was 77% at 5.0 BV. This was similar to the results of Yang, et al using macroporous resin to deproteinization of crude polysaccharide from pumpkin residues (Yang et al., 2012). According to static desorption experiments, 60% ethanol was used for dynamic desorption. The research showed that protein was almost completely desorbed at 3.0 BV, while polysaccharide was completely desorbed at 2.0 BV. Therefore, the processing volume of HC‐CPS solution on HP‐20 was approximately 1.5 BV. In this condition, the deproteinization ratio was 91% and the polysaccharide recovery ratio was 62%.

### 3.5 Comparison of the purification efficiency by HP‐20 resin and three traditional methods

Table 6 shows the purification effects of HP‐20 resin, trichloroacetic acid, Sevage reagent, and activated carbon for rapeseed meal polysaccharides, respectively. It was observed that the deproteinization ratio of HP‐20 resin was much better than the three traditional methods and the polysaccharide recovery ratio of HP‐20 resin was slightly lower than three traditional methods. The trichloroacetic acid and Sevage reagent was denatured protein and then centrifuged to remove precipitated protein. The mild conditions of Sevage method did not destroy the polysaccharide structure, but the poor deproteinization effect usually repeated many times to completely remove the protein. The trichloroacetic acid method usually caused some of the polysaccharide structure to be destroyed. Therefore, the polysaccharide recovery ratio of the Sevage method was higher than that of the trichloroacetic acid method. This HP‐20 resin was a polymeric adsorbent with large internal surface areas and had a much more consistent structure compared with active carbon (Kammerer, Carle, Carle, & Kammerer, 2011), which resulted in a stronger adsorption ability of HP‐20 resin. Therefore, the deproteinization effect of HP‐20 resin was better than that of active carbon, but the polysaccharide recovery ratio was lower than that of active carbon. Consequently, the purification of rapeseed meal polysaccharides with HP‐20 resin was better than the three traditional methods.

**Table 6:** Different purification methods influencing deproteinization ratio and polysaccharide recovery ratio

Methods	Deproteinization ratio	Polysaccharide recovery ratio
HP−20 resin	82 ± 9%	87 ± 1%
trichloroacetic acid	35 ± 1%	88 ± 5%
Sevage reagent	13 ± 1%	98 ± 2%
active carbon	16 ± 5%	97 ± 1%

### 3.6 Characterization of polysaccharide before and after adsorption

**Figure 5:**
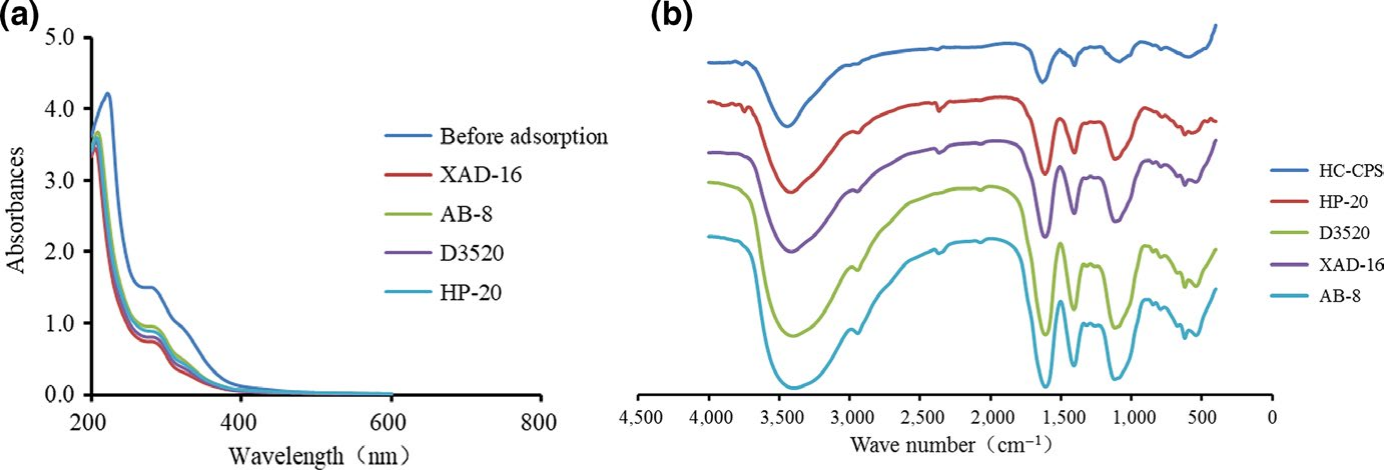
The UV/vis spectra and FT‐IR spectra of the polysaccharide before and after adsorption

To verify the effectiveness of the resin treatment, the samples were subjected to UV/vis scanning and FT‐IR spectroscopy. As shown in Figure 5a, the absorbance in the region of 200–600 nm was decreased (Shi et al., 2017), especially between 200 and 400 nm. It indicated that most protein was adsorbed by this resins. The FT‐IR spectra of the samples before and after treatment with four resins are shown in Figure 5b. The band between 3,500 and 3,300 cm^−1^ was due to OH stretching (Cobs‐Rosas, Concha‐Olmos, Concha‐Olmos, Weinstein‐Oppenheimer, & Zuniga‐Hansen, 2015). The two bands at 1,590 and 1,420 cm^−1^ corresponded to the asymmetric and symmetric vibration of the COO (carboxylate) structure (Luan, Wu, Zhang, & Wang, 2012). The several peaks in the region of 1,200–950 cm^−1^ were assigned to the vibrations of glycosidic bonds and pyranoid rings, which was a typical region for polysaccharides (Wei et al., 2018). Comparing the spectrum before and after the treatment of the four resins, the significant increase in absorption at 1,200–950 cm^−1^ indicated that the purity of the polysaccharide after resin treatment was significantly improved. According to previous reports, the resin adsorption did not destroy the molecular structures of polysaccharide (Cobs‐Rosas et al., 2015; Yang et al., 2012; Zhu & Wu, 2009).

## 4 CONCLUSIONS

In the study, the deproteinization of HP‐20, D3520, XAD‐16, and AB‐8 resins were systematically investigated by adsorption and desorption experiments. The pseudo‐second‐order model and Freundlich model were more suitable models for characterizing the adsorption behavior of four resins. The resin adsorption did not destroy the molecular structures of polysaccharide. The HP‐20 resin had high deproteinization ratio and was suitable for deproteinization of rapeseed meal polysaccharides. This study provides new insights into purifying the polysaccharides extracted from rapeseed meal by HP‐20 resin. This achievement may also be helpful to the comprehensive utilization of rapeseed meal.

## CONFLICT OF INTEREST

The authors declare that they do not have any conflict of interest.

## ETHICAL APPROVAL

This study does not involve any human or animal testing.

## INFORMED CONSENT

Written informed consent was obtained from all study participants.
